# Arachidonic Acid Pathways and Male Fertility: A Systematic Review

**DOI:** 10.3390/ijms24098207

**Published:** 2023-05-03

**Authors:** Malvina Hoxha, Arcangelo Barbonetti, Bruno Zappacosta

**Affiliations:** 1 Department for Chemical-Toxicological and Pharmacological Evaluation of Drugs, Faculty of Pharmacy, Catholic University Our Lady of Good Counsel, 1000 Tirana, Albania; 2 Andrology Unit, Department of Life, Health and Environmental Sciences, University of L’Aquila, 67100 L’Aquila, Italy

**Keywords:** male fertility, arachidonic acid pathway, prostaglandins

## Abstract

Arachidonic acid (AA) is a polyunsaturated fatty acid that is involved in male fertility. Human seminal fluid contains different prostaglandins: PGE (PGE_1_ and PGE_2_), PGF_2α_, and their specific 19-hydroxy derivatives, 18,19-dehydro derivatives of PGE_1_ and PGE_2_. The objective of this study is to synthesize the available literature of in vivo animal studies and human clinical trials on the association between the AA pathway and male fertility. PGE is significantly decreased in the semen of infertile men, suggesting the potential for exploitation of PGE agonists to improve male fertility. Indeed, ibuprofen can affect male fertility by promoting alterations in sperm function and standard semen parameters. The results showed that targeting the AA pathways could be an attractive strategy for the treatment of male fertility.

## 1. Introduction

**Figure 1:**
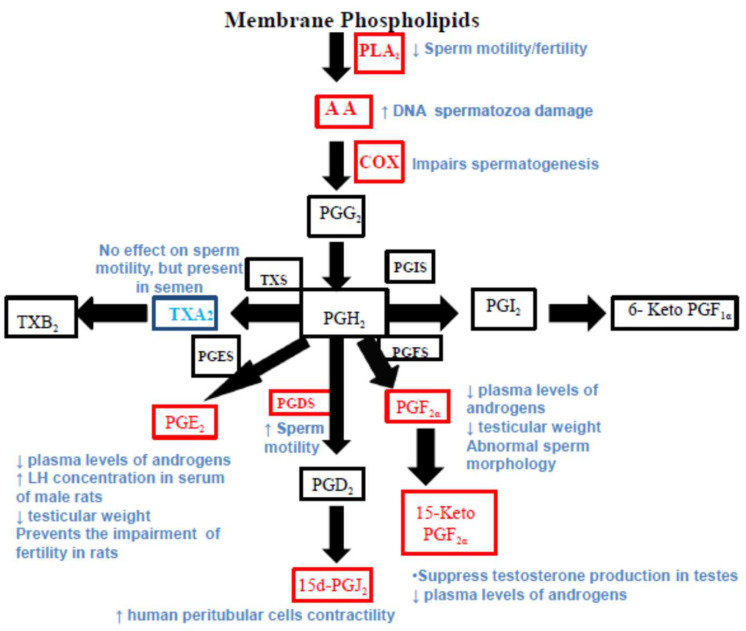
Arachidonic acid pathway and the main metabolites/enzymes involved in male fertility. Arachidonic acid mediators or enzymes that are marked in red are known to be involved in male fertility. Abbreviations: arachidonic acid (AA), phospholipase A_2_ (PLA_2_), cyclooxygenase (COX), prostaglandin G_2_ (PGG_2_), prostaglandin H_2_ (PGH_2_), thromboxane synthase (TXS), thromboxane A_2_ (TXA_2_), thromboxane B_2_ (TXB_2_), prostaglandin E synthase (PGES), prostaglandin E_2_ (PGE_2_), prostaglandin D synthase (PGDS), prostaglandin D_2_ (PGD_2_), 15-deoxy-D12,14-prostaglandin J_2_ (15d-PGJ_2_), prostaglandin F synthase (PGFS), prostaglandin F_2α_ (PGF_2α_), 15-keto Prostaglandin F_2α_ (15-keto PGF_2α_), prostaglandin I synthase (PGIS), prostacyclin (PGI_2_), 6-keto Prostaglandin F_1α_ (6-keto PGF_1α_).

Arachidonic acid (AA) is a polyunsaturated fatty acid released by the activation of phospholipase A_2_ (PLA_2_) that is transformed into a series of metabolites by different enzymes. Reactive oxygen species (ROS) and cytokines can also activate PLA_2_. The most known pathway of AA is the cyclooxygenase (COX) pathway which is responsible for the production of prostaglandins (PGs) and thromboxane (TX). The lipoxygenases (LOs) pathway brings to the production of both, leukotrienes (LT) and anti-inflammatory lipoxins (LXs) (Figure 1) [1]. Cytochrome P450 (CYP) enzyme is another pathway of AA transformation that gives rise to epoxyeicosatrienoic acids (EETs), and 20-hydroxyeicosatetraenoic acid (20-HETE). 5-, 8-, 12-, and 15-lipoxygenases (15-LOX) produce hydroperoxyeicosatetraenoic acid (HPETE).

Von Euler in the earliest 1930s was the first to report the presence of PGs in the seminal plasma. They were thought to originate from the prostate gland, and for this reason they were named prostaglandins [2]. The PGs present in human semen are the PGE (PGE_1_ and PGE_2_), PGF_2α_, and their specific 19-hydroxy derivatives, 18,19-dehydro derivatives of PGE_1_ and PGE_2_. PGE_2_ and 19-hydroxy PGE (19-OH PGE) are the main PGs present in semen [3,4]. PGs content in fertile men’s ejaculate is 1 mg [5]. Seminal vesicles are the main source of PGs in human semen [6], with additional contributions from epididymis, vas deferens, and testes [7]. Ollero et al. reported that high levels of AA are related to defective human spermatozoa [8,9]. In line with these findings, other studies demonstrated that the exposure of human spermatozoa to AA and other PUFAs can cause DNA spermatozoa damage [10]. Cosentino et al. showed that the levels of PGE_2_ are 15 to 25 times higher in the lumen of ram cauda epididymidis in respect to rete testis. In washed sperm recovered from the vas deferens of rams PGE_2_ increases the sperm cyclic adenosine monophosphate (cAMP) [7]. It is still unclear whether this is a receptor-mediated process. Interestingly Hedqyist et al., reported that PGs can also mediate the uptake of Ca^2+^ into the cell [11]. PGE can decrease calcium uptake in spermatozoa through the increase of intercellular cAMP concentration [12]. Another potential role of PGs can be the sperm transport through the distal epididymidis and vas deferens [13]. PGD_2_ can regulate epithelial apoptosis [14]. Lipocalin type prostaglandin D-synthase (L-PGDS) that is responsible for the production of prostaglandin D_2_ (PGD_2_) is abundant in the seminal fluid of fertile men, and is significantly decreased in the semen of oligozoospermic men [15]. The role of seminal L-PGDS is to provide retinoids in seminiferous tubules, as well as the maturing spermatozoa in the epididymis [16]. L-PGDS is found either in seminal plasma, or on sperm surface, and L-PGDS levels were significantly reduced in oligozoospermic patients. Chen et al. reported that L-PGDS may act as the main protein in improving the progressive motility of sperm by increasing either the capacity of sperm to bind to eggs or to agglutinate, or by increasing the percentage of motile sperm [17]. In addition, COX-2 was found to be implicated in testicular inflammation related to idiopathic infertility in biopsies of men with impaired spermatogenesis [18]. Alteration of PGs synthesis in the testis is another potential reason for idiopathic male fertility [19]. However, both COX-1, and COX-2 isoforms were not found in normal human testes [20]. Schell et al. demonstrated that testicular PGD synthases are expressed in normal and pathological situations [21]. There are few reports on the role of 15-Hydroperoxyeicosatetraenoic acid (15-HETE) in sperm function and membrane integrity [22]. 15(S)-lipooxygenase was figured out to be involved in sperm acrosome reaction [23,24]. The aim of this study is to synthesize the existing evidence on the implication of PGs in male fertility.

## 2. Methods

### 2.1. Research Methods and Reporting

This systematic review followed the preferred reporting items for systematic reviews (PRISMA) guidelines.

### 2.2. Study Design

We conducted a systematic review to report all the findings of in vivo animal studies and human clinical trials on the association between AA pathways and male fertility.

### 2.3. Eligibility Criteria

The eligibility criteria for inclusion were: All Randomized Controlled Trials (RCTs), or observational studies (cohort or case control design) reporting the association of the AA pathway with male fertility. We excluded reviews, and studies on the association of AA pathways with female fertility. The literature search was not restricted by the year of publication.

### 2.4. Literature Search, Information Sources and Selection of Articles

We collected all the relevant data that conformed to the eligibility criteria on the role of the AA pathway in male fertility. We searched in Pubmed, Scopus, Medline, and Embase databases all studies reporting the association between arachidonic acid mediators and male fertility using the following text words; “arachidonic acid and male fertility”; “prostaglandin and male fertility”; “thromboxane and male fertility”; “leukotriene and male fertility”;“lipoxin and male fertility”; “5-lipoxygenase and male fertility”; “12-lipoxygenase and male fertility”; “15-lipoxygenase and male fertility”; “pro-resolving lipid mediators and male fertility”; “cytochrome P450 epoxygenase pathway and male fertility”. Only studies that fulfilled the eligibility criteria were included.

### 2.5. Data Synthesis

Only 68 articles out of 456 identified articles, were included in this systematic review.

Duplicates articles, studies that did not report the association of AA pathways with male fertility, reviews, and original articles reporting AA pathways and female fertility were excluded.

The findings were classified in three tables, reporting findings from animal studies, human studies, and studies on the potential effects of NSAIDs in seminal PGs. One article was placed in more than one table because it contains data from animal study, and data on the effects of NSAIDs (flurbiprofen, indomethacin) in seminal prostaglandins.

## 3. Results

**Figure 2:**
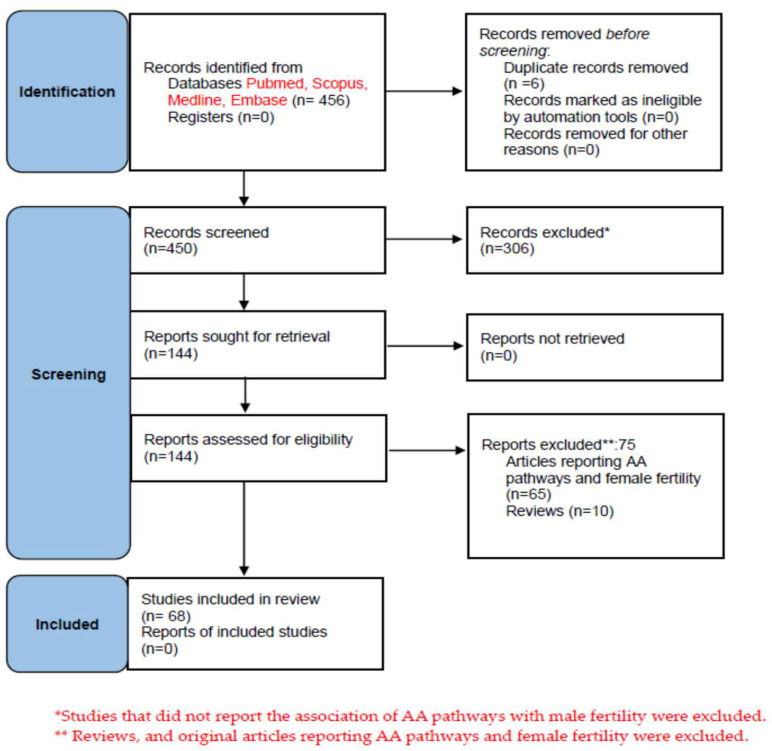
Prisma Flow diagram of literature search and selection for articles included in this systematic review.

A schematic diagram of the literature search procedure is shown in Figure 2.

### 3.1. Findings from Animal Studies

As shown in Table 1, we identified a total of 17 studies carried out in different animal models (male rats, mice, dromedary camels, ram/bulls). Different AA mediators or enzymes involved in the AA pathway were assessed (AA, PGE_1_, PGE_2_, PGF_2α_, sPLA_2_, PGDS).

A study conducted in dromedary camels showed that sPLA_2_ is a fertility-associated biomarker in seminal plasma and serum. Group VIA Phospholipase A_2_ (iPLA_2β_) has an important role in spermatozoa [25]. Data showed also that prostaglandin D synthase (lipocalcin-type) is negatively related to camels’ fertility [26]. Fouchécourt et al. showed that prostaglandin D synthase had a high capacity to bind to testosterone and its levels were increased in animals with normal or high fertility [27].

Higher levels of AA were found in Acsl6 (long-chain acyl-CoA synthetase 6) knockout (*Acsl6*^−/−^) male mice testes [28]. *Acsl6* knockout (*Acsl6*^−/−^) male mice were severely subfertile with reduced sperm counts and smaller testes [28].

AA is one of the main components of testicular membranes in mice [29]. In addition, AA was found to be not as effective as DHA in restoring fertility and sperm count [30]. The higher absolute amount of n-3 and n-6 PUFA are more important for reproduction than the n-6/n-3 FA ratios [31].

PGE_2_ is the main PG found in the seminal fluid [32]. Different studies reported that intravenous or intraventricular administration of low dose PGs of the E series in male rats can enhance the production of LH [33,34]. Interestingly intratesticular injection of 2.5 mg/kg of PGE_l_, or PGE_2_ affects the capacity of fertilizing of epididymal spermatozoa in male rats. Instead, no effects on male rat fertility were reported for PGF_2α_, despite suppressing the testis and epididymis weight [35]. Both PGs of the E series (PGE_1_ and PGE_2_) did not have any local effect on epididymal spermatozoa fertilizing capacity, despite reducing the weight of the injected testis due to blood flow restriction at the site of application. Considering that PGs suppress steroidogenesis by lowering the plasma levels of androgens, the incapacity of PGs to affect the fertilizing capacity of spermatozoa in both injected and contralateral sides suggests that the androgen that has been available following the treatment schedule was enough to keep the fertilizing capacity of the epididymal spermatozoa despite of the PGs site of injection [35]. However, intratesticular injection of higher doses of PGs (2.5 mg/kg) adversely affected the fertilizing ability of spermatozoa, by affecting steroidogenesis in both the injected and contralateral control testes [35]. The short-term treatment of hamsters with PGF_2α_, or PGE_1_ had no effect on fertilizing capacity [36,37]. Testosterone added concurrently with PGE_1_ and PGE_2_ maintained both the fertilizing capacity of epididymal spermatozoa, and the weight of accessory sex glands [38]. Hafiez et al. hypothesized that PGE_2_ in testes can modify the effect of trophic hormones and prevent the impairment of fertility in rats [39]. The plasma levels of androgens are decreased by both PGE_2_ and PGF_2α_ [33]. 15-Me-PGF_2α_ can suppress testosterone production in the testes [40].

The testicular regression observed in the injected testis (PGE_1_, PGE_2_, PGF_2α_), could be explained by the restricted blood flow through the testes following the local application of PGs [41].

**Table 1:** Overview of the main outcomes of animal studies included in this systematic review.

No.	Study	Type of Animal	AA Pathway Mediator/Enzyme Assessed	Results
1	Castracane et al., 1974 [33]	Male Rats	PGE_1_, PGE_2_	A single PGE_2_ (sub-q injection) increases gradually the LH concentration in serum of river male rats of known fertility.
2	Rej et al., 1980 [35]	Male Rats	PGE_1_, PGE_2_, PGF_2α_	The incapacity of PGs (1 mg/kg of PGE(1 and 2) and PGF_2α_) to affect the fertilizing capacity of spermatozoa in both injected and contralateral sides suggest that androgen that has been available following the treatment schedule was enough to keep the fertilizing capacity despite of the PGs site of injection.
3	Ericsson et al., 1973 [38]	Male Rats	PGE_1_, PGE_2_	Testosterone added concurrently with PGE_1_ and PGE_2_ maintained both the fertilizing capacity of epididymal spermatozoa, and the weight of accessory sex glands.
4	Bartke et al., 1973 [36]	Mice	PGF_2α_	PGs can suppress steroidogenesis in the testis and this can bring to a decrease in plasma testosterone levels in intact adult male mice.
5	Kimball et al., 1978 [40]	Male Rats	15-Me-PGF_2α_	15-Me-PGF_2a_ can suppress testosterone production in testes.
6	Memon, 1973 [34]	Male Rats	PGE_2_, PGF_2α_	The testicular weight and plasma testosterone is lower in rats treated with PGE_2_, and PGF_2α._. LH increased significantly in male rats with bilateral injection of PGs.
7	Free et al., 1972 [41]	Rats	PGE_1_, PGE_2_, PGF_2α_	PGE_1_, PGE_2_, PGF_2α_ can lower the blood flow through the testes. No further data were reported on fertility.
8	Lubice-Nawrocki et al., 1973 [37]	Hamster	PGE_1_, PGF_2α_	The treatment of hamsters for a short period of time with PGF_2α_, or PGE_1_ had no effect on fertilizing ability.
9	Gerozissis et al., 1981 [32]	Rats	PGE_2_	PGE_2_ was found in the seminal fluid of rats, but no further data were reported on sperm fertility (motility, morphology, etc.).
10	Waheed et al., 2015 [26]	DromedaryCamels	sPLA_2,_ PGDS	sPLA_2_ is a fertility-associated biomarker in seminal plasma and serum of dromedary camels. Prostaglandin D synthase (lipocalcin-type) is negatively related to camels’ fertility.
11	Roqueta-Rivera et al., 2010 [30]	Male Mice	AA	AA was not as effective as DHA in restoring fertility, sperm count, and spermiogenesis in male mice.
12	Hale et al., 2019 [28]	Male mice	AA	Higher AA levels were found in Acsl6 ^−/−^ testes.
13	Stoffel et al., 2020 [29]	Mice	AA	AA is one of the main components of testicular membranes. Only the AA/DHA (1:1 M) diet fully restored male spermatogenesis in the ω6/ω3- fatty acid desaturase (fads2^−/−^) cohorts.
14	Bao et al., 2004 [25]	Male Mice	PLA_2_	Group VIA Phospholipase A_2_ (iPLA2β) reduces the motility of spermatozoa.
15	Khatibjoo, 2018 [31]	BroilerBreeder	AA	Higher absolute amount of n-3 and n-6 PUFA are more important for the reproductive and performance traits of breeders than the n-6/n-3 FA ratios.
16	Hafiez, 1974 [39]	Rats	PGE_2_	PGE_2_ prevented the impairment of fertility in rats.
17	Fouchécourt et al., 2002 [27]	Ram/Bull	PGDS	PGDS levels were increased in animals with normal or high fertility, and showed a high capacity to bind to testosterone.

### 3.2. Summary of the Results Reported by Human Clinical Trials

In Table 2, we report the main findings of human clinical studies (31 studies) that were systematically reviewed.

Isidori et al. reported that PGs can be either reduced or increased in infertile men, hence either high or low levels of PGs can be harmful [42].

PGE, PGF, and 19-OH PGF, 19-OH PGE, 6-keto-PGF_1α_, and PGD_2_, were detected in the semen of fertile men [5,34,43,44]. Rather unexpectedly higher levels of PGE_2_, PGF_2α_, PGI_2_, and TXA_2_ were observed in the seminal plasma of diabetic patients [45], considering that diabetes is known to significantly reduce fertility rates. Despite increased seminal plasma PG concentrations are associated with oligospermia and reduced sperm motility the current data did not show these sperm defects in diabetic males [45]. Different evidence reports a positive correlation between AA levels and spermatozoa [46]. Higher levels of AA were detected in the semen of eighty-two infertile men with idiopathic oligoasthenoteratozoospermia (OAT) compared to the semen of 78 fertile men [47]. A statistically significant negative correlation was revealed between the ratios of AA with docosahexaenoic acid (DHA), and eicosapentaenoic acid (EPA), and the total sperm number, morphology, and motility [47]. Hawkins states that the higher the concentrations of semen PGs, the lower the number of abnormal spermatozoa [48]. Consentino et al. reported that PGF_2α_ was correlated with abnormal sperm morphology. In addition, the data revealed that Zn^2+^ and Ca^2+^ concentration were predictors of seminal PGF_2α_, instead of sperm motility, plasma testosterone, and Ca^2+^ concentration which were significant predictors of seminal PGE [13]. High levels of seminal PGF_2α_ may be related to impaired spermatogenesis [7].

However, other evidence has shown that the semen of fertile men contains higher levels of PGs with respect to semen of infertile men [49,50]. 19-OH-PGE and PGE levels were significantly decreased in the semen of 10 infertile males [49]. In addition, it was found that PGs levels in the semen of hypogonadal men were correlated with testosterone concentration in the blood [51]. Specifically, low PGE levels have been found either in the semen of men with “idiopathic” infertility, or of men of infertile marriages where no abnormal findings were revealed, pointing to the essential role of PGE in sperm functions not assessable by standard semen analysis [52,53,54]. In line with these findings, other data showed higher levels of PGE_2_ and 19-OH PGE in the seminal plasma of fertile men with respect to the seminal plasma of patients with OAT [55]. Reduced levels of PLA_2_ were observed in patients with globozoospermia [56]. Isidori et al. showed that the reduced adenylcyclase and testicular androgen activity may be responsible for the negative impact of low seminal PGs levels on sperm concentration and motility [42]. Indeed, reduced sensitivity of receptors to increased titers of PGs, and DNA synthesis inhibition in testes may be responsible for the negative impact of high seminal PGs levels.

In a Swedish study, it was revealed that 19-hydroxy PGF and 19-hydroxy PGE have a significant role in sperm motility, potentially through the ATP content in spermatozoa [57]. 15-deoxy-Δ12-14-PGJ_2_, a product of COX/PGD synthase, could be involved in human sub-/infertility by affecting the human peritubular cells contractility and phenotype potentially through ROS [58].

PGD_2_ synthetases (PGDS) were found in the interstitial cells of men with impaired spermatogenesis [21]. Chen et al. showed that lipocalin type (L-PGDS) in seminal plasma is positively correlated with sperm motility and density [17].

F_2_-isoprostanes (F_2_-IsoPs) are produced by the oxygenation of AA, and are related to male infertility, as a marker of sperm immaturity by affecting sperm quality [59,60]. In infertile patients with varicocele, it was observed a positive correlation between F_2_-IsoPlevels and sperm immaturity [60,61]. Recently Moretti et al., assessed the cut off value of F_2_-IsoPs semen levels in fertile and infertile men, and reported that F_2_-IsoP levels above 29.96 ng/mL are potentially related to idiopathic infertility and other pathological conditions, and can serve as an index of altered sperm quality in infertile patients [62]. Instead, resolvins are AA specialized proresolving mediators, and specifically RvD1 is an indicator of seminal pathological state [63]. High levels of RvD1 are associated with changes in sperm parameters, hypothesizing that resolvins cannot defend the male fertility, in the presence of chronic inflammation. These data support the fact that diets rich in n-3 PUFA can be helpful in male infertility with an inflammatory state [63].

However other controversial studies demonstrated no correlation between seminal PGs concentration and sperm morphology, motility, concentration, and fertility [64,65]. Dorp et al. revealed no correlation between seminal PGs and spermatozoa morphology and concentration [66]. Templeton et al. reported no significant difference in semen PGE levels between fertile and infertile men [4]. These data were further confirmed by Schlegel’s findings, where no decrease in the PGE concentration was detected either in the semen of fertile, and non-fertile men [67].

**Table 2:** Overview of the characteristics of human clinical studies included in the systematic review.

No.	Study	Sample Size and Characteristic/Age Range or Mean Age	AA Mediators Studied	Results
1	Skakkeback et al., 1976 [51]	Semen samples of 2 hypogonadal men/26 and 36 years old respectively	PGE_1_, PGE_2_, 19-OH-PGEs	The seminal 19-OH PGEs levels change based on blood testosterone levels, and may be involved in reproductive process.
2	Cosentino et al., 1984 [13]	163 semen samples from 145 men/24 to 50 years old	PGF_2α_, PGE	PGs levels in the semen are essential for assessing the human male fertility and are related to certain male fertility parameters. Patients with low seminal PGE levels also had reduced sperm motility. High levels of seminal PGF_2α_ may be related to impaired testicular function. Seminal PGF_2α_ is positively correlated to abnormal sperm morphology.
3	Lewy et al., 1979 [49]	Semen of 10 infertile males and 7 fertile males/N.S.	19-OH-PGE, PGE, 6-keto-PGF_1α_	Lower levels of PGE and 19-OH-PGE were found in the infertile group in respect to the fertile men. 6-keto F_1α_ levels in human semen did not change, and PGI_2_ is not important for male fertility.
4	Bygdeman et al., 1970 [52]	Single semen samples from 150 men, classified in 3 classes: (Group A, fertile men (*n* = 29); Group B, men in noninvestigated infertile marriages (*n* = 100); and Group C, men in infertile marriages with no abnormal clinical or laboratory findings (*n* = 21)/N.S.	PGE_1_, PGE_2_,PGE_3_, PGA_1_, PGA_2_,PGB_1_,PGB_2_, 19-hydroxy PGA_1_, 19-hydroxy PGA_2_, 19-hydroxy PGB_1_, 19-hydroxy PGB_2_	The PGE levels in the seminal plasma have an essential role in male fertility. Seminal PGE, increase the human female reproductive tract uterine contraction.
5	Collier et al., 1975 [53]	12 men in infertile marriages with no abnormal findings/N.S.	PGE	PGE levels in the semen of infertile men is lower than semen of fertile men, but no abnormality on sperm motility had been detected by semen analysis.
6	Kelly et al., 1979 [65]	57 semen samples/20–40 years old	PGE_1_, PGE_2_, 19-OH PGE_1_, 19-OH PGE_2_	In the semen samples of polyzoospermic group the PGs levels decreased significantly. Reduced PGs levels in polyzoospermia may suggest that the function of seminal PGs is to modify sperm metabolism at the time of ejaculation.
7	Asplund, 1947 [64]	155 specimens of sperm/N.S.	PGs	No correlation between seminal PGs concentration and sperm morphology, motility and concentration exists.
8	Hawkins et al., 1961 [48]	*n* = 4 normal human testicular samples, *n* = 13 pathological samples, *n* = 6 additional samples/26 to 43 years old	PGs	The higher the spermatozoa percentage the lower the number of PGs. However, in a specific group of infertile patients, higher concentration of PGs were related to abnormal sperm motility. 15dPGJ_2_ is potentially involved through ROS, in enhancing hypertrophy and deprivation on the contractility of peritubular cells from human testes and is potentially involved in development of human male sub-/infertility.
9	Horton et al., 1964 [50]	14 semen samples/N.S.	PGE_1_	PGs concentration in human semen varied from 24 to 783μg/mL. No data were reported on male fertility.
10	Brummer et al., 1972 [54]	104 samples divided in 4 groups/N.S.	PGE, PGA	Lower levels of PGE were observed in the samples of seminal fluid of infertile men. High PGE levels may increase fertility, through the increase of sperm count and motility.
11	Huleihel et al., 1999 [55]	17 samples divided in 2 groups Fertile men (*n* = 7), versus patients with oligoteratoasthenoazoospermia (OTA) (*n* = 10)/N.S.	PGE_2_	Higher levels of PGE_2_ were observed in the seminal plasma of fertile men in respect to seminal plasma of patient with oligoteratoasthenoazoospermia (OTA), but no differences in sperm cells functions and parameters were observed.
12	Isidori et al., 1980 [42]	15 normal volunteers, and 30 patients with PGE seminal levels inferior to normal minimal values; 8 patients with seminal PGE levels greater than normal maximal values; 22 patients with seminal 19-OH PGE levels inferior to normal minimal values; 16 patients with seminal 19-OH PGE levels greater than normal maximal values/N.S.	PGE, 19-OH PGE	The reduced adenylcyclase and testicular androgen activity may be responsible of the negative impact of low seminal PGs levels in sperm concentration and motility. Indeed, reduced sensitivity of receptors to increased titers of PGs, and DNA synthesis inhibition in testes may be responsible of the negative impact of high seminal PGs levels.
13	Sturde, 1968 [44]	Semen samples from volunteers/N.S.	19-OH PGF, 19-OH PGF_2α_, 19-OH PGE_2_, PGE_1_, PGE_2_, PGF_2α_	19-hydroxy PGF and 19-hydroxy PGE have a significant role in sperm motility, potentially through the ATP content in spermatozoa.
14	Schell et al., 2007 [21]	Normal patients with obstructive azoospermia (*n* = 6), and impaired spermatogenesis (*n* = 8)/N.S.	PGD_2_ synthetases	PGD_2_ synthetases are found in interstitial cells of men with impaired spermatogenesis.
15	Gerozissis et al., 1981 [34]	Semen of fertile men/30–50 years with a proven fertility (children 3–5 years of age).	19-OH-PGE, 19-OH-PGF_α_PGD_2_, PGE_2_, PGE_1_, PGF_2α_, PGF-_1α_, 6-keto-PGF_1α_ 13,14-dihydro-15-keto-PGF_α_	19-OH-PGE, 19-OH-PGF_α_,PGE_2_, PGE1, PGF_2α_, PGF-_1α_, 6-keto-PGF_1α_, PGD_2_, 13,14-dihydro-15-keto-PGF_α_ were found in human semen of fertile men.
16	Templeton et al., 1978 [4]	Semen of 23 fertile men/20–40 years old	PGE, 19-OH PGE, PGF, 19-PGF	No significant difference was showed in the PGE levels in the semen of fertile and non-fertile men. The sperm count and motility were normal.
17	Tusell et al., 1980 [43]	Semen of 7 volunteers/25–30 years old	19-OH PGE, 19-OH PGF, PGE	PGE, PGF, and 19-hydroxylated E and F were detected through gas and liquid chromatography in semen of fertile men.
18	Bendvold et al., 1987 [5]	Semen of 31 men/N.S.	PGE, PGF, 19-hydroxy-PGE, 19-hydroxy-PGF	A positive correlation exists between PG content and sperm density in fertile men. PGE and 19-hydroxy-PGE were the main PGs in human semen.
19	Schlegel et al., 1981 [67]	Semen of 10 fertile and 55 infertile men/N.S.	PGE_2_, PGF_2α_	PGF_2α_ can act on sperm motility, but not through its receptors. High levels of PGE were found in patients with persisting varicocele and in patients with very poor motility and low sperm counts.
20	Signorini et al., 2022 [63]	Infertile Italian patients (*n* = 67) versus fertile men (*n* = 18)/29 to 40 years old	Resolvin D_1_; F_2_-IsoPs	Resolvin D_1_ levels increase in patients with idiopathic infertility, leukocytospermia, varicocele in respect to fertile men. Resolvin D_1_ and F_2_-IsoPs reduce the sperm quality. Resolvin D1 levels correlated negatively with sperm progressive motility, vitality, fertility index; but positively with sperm necrosis and immaturity.
21	Longini et al., 2020 [59]	Semen samples of 61 Italian men/27–42 years old	F_2_-dihomo-IsoPs, F_2_-IsoPs, F_4_-NeuroPs	F_2_-IsoPs are related to male infertility by affecting the sperm quality. F_2_-IsoPs showed a negative correlation with sperm motility and a positive one with sperm immaturity.
22	Safarinejad et al., 2010 [47]	82 males/34.2 ± 4.1 years old	AA	Higher levels of AA were detected in infertile men compared to fertile men. A strong negative correlation was found between the AA:DHA and AA:EPA ratios and total sperm count, sperm motility, and sperm morphology.
23	Shrivastav et al., 1989 [45]	18 randomly selected IDDM male diabetic patients/mean age 31 years, (age range 21–39 years)	PGE_2_, PGF_2α_, PGl_2_,TXA_2_	In diabetic patients, higher levels of PGE_2_, PGF_2α_, PGl_2_, TXA_2_ were observed in seminal plasma. Despite increased seminal plasma PG concentrations are associated with oligospermia and reduced sperm motility the current data did not showed these sperm defects in the diabetic males.
24	Collodel et al., 2021 [60]	49 infertile couples/29–37 years	F_2_-IsoPs	F_2_-IsoPs can be a marker of sperm immaturity, for the evaluation of semen and follicular fluid quality.
25	Andersen et al., 2016 [46]	144 samples/≥18 years old	AA	A positive correlation between AA levels and spermatozoa was observed.
26	Collodel et al., 2018 [61]	38 patients/26–40 years	8-Iso PGF_2α_	A significant positive correlation between F_2_-IsoP levels and sperm immaturity was observed in infertile patients with varicocele.
27	Chen et al., 2007 [17]	90 semen samples/N.S.	L-PGDS	L-PGDS in seminal plasma is positively correlated with sperm motility and density.
28	Moretti et al., 2019 [56]	1 patient/44 years old	PLA_2_	Reduced levels of PLA_2_ were observed in sperm of globozoospermic patient respect to those of fertile men.
29	Moretti et al., 2022 [62]	147 patients with infertility/26–43 years	F_2_-IsoPs	F_2_-IsoP levels above 29.96 ng/mL are potentially related to idiopathic infertility and other pathological conditions and is an index of altered sperm quality in infertile patients.
30	Gerozissis et al., 1982 [7]	Men with proven fertility; Vasectomized men; Men with an obstructive sterility/N.S.	PGE_1_, PGE_2_, PGF_1α_,PGF_2α_,19-OH-PGE (1 + 2), 19-OH-PGF_α_	The major part of PGs in human semen derive from seminal vesicles. High levels of seminal PGF _2α_ may be related to impaired testicular function.
31	Dorp et al., 1968 [66]	Semen samples/N.K.	PGs	No correlation was found between seminal PGs and spermatozoa morphology and concentration.

### 3.3. Potential Effects of NSAIDs in Seminal Fluid PGs

The main characteristics of the NSAIDs’ role in seminal fluid PGs and male fertility that were systematically reviewed are summarized in Table 3.

Bendvold et al. showed that all PGs (PGE, PGF, 19-hydroxy-PGE, and 19-hydroxy-PGF,8α-19-hydroxy-PGF_2α_, 8ß-19-hydroxy-PGF_1α_) were reduced in semen samples of six volunteers before, during and after treatment with naproxen 250 mg 3 times daily for 2 weeks [68]. Interestingly, the results showed that for maintaining normal sperm motility it is essential to have a balanced concentration ratio between 19-hydroxy-PGF and 19-hydroxy-PGE. The short treatment with naproxen did not have any impact on human fertility. The data suggest that the reduction of PGs is not secondary to the effect of naproxen on sperm characteristics [68]. However further studies need to be performed to assess the long-term effect of naproxen, or other NSAIDs on fertility and sperm characteristics (motility, morphology, density).

Other studies using Aspirin showed a significant reduction of PGE_2_ and PGF_2α_ levels in human seminal fluid during treatment with aspirin [69], and an 80% of reduction of seminal plasma levels of PGE following treatment with 7.2 g/day of Aspirin [70]. The mechanisms responsible for controlling the concentrations of PGE_2_ and PGF_2α_ in semen may be different [70]. The subchronic dose of aspirin (12.5 mg/kg for 30 days and 60 days) given to male rats reduced sperm mobility and density [71]. These data were confirmed in other animal models where sperm motility, morphology and seminal volume were decreased, and spermatogenesis was impaired following aspirin use [72,73,74,75,76]. In humans, moderate aspirin use reduced the motile, progressive and rapid progressive gametes percentages [77], having a negative impact on male fertility [78]. Interestingly, the fertility increased in male mice that were classified initially as sub-fertile under treatment with aspirin (50 mg/kg twice daily for a total of 12 days) [79]. Other studies did not conclude on the role of Aspirin on spermatogenesis [80].

A recent study carried out in male rats that received ibuprofen (0; 2.4; 7.2 or 14.3 mg/kg/day) showed that the pre-pubertal treatment with ibuprofen had a negative effect on sperm quality and quantity, which affects reproduction [81]. The male offspring had an accelerated sperm transit time in the epididymis, while the fertility potential was reduced in the female offspring [81]. However, Stutz et al. showed that intramuscular injection of ibuprofen 5.6 mg/kg day reduced testosterone levels, but did not modify the sperm functional activity [82]. Flurbiprofen and indomethacin did not affect male reproduction in rats [27]. However other study showed that flurbiprofen produces a small alteration in sperm head with a larger and spherical head [83]. Löscher et al. reported that chronic treatment with phenylbutazone may improve sperm quality, increase ejaculate volume, and improve sperm fertility [84].

NSAIDs (indomethacin, naproxen, acetylsalicylic acid) decreased the PGE_2_ levels in seminal fluid in rats, suggesting that these compounds can be used to control male fertility [85]. The reduction of prostaglandin synthesis in male rats does not have any effect on fertility. This can be explained by the very low seminal prostaglandin levels in rats in confront to other animals [85].

Interestingly, Conte et al., showed that indomethacin improved significantly the sperm count and motility in infertile oligozoospermic patients with high levels of PGs [86]. The number of pregnancies was reduced in the groups mated with indomethacin and oxyphenbutazone treated male rats [87].

**Table 3:** Potential Effects of NSAIDs in seminal fluid prostaglandins.

No.	Study	Type of Study/Sample Characteristics	Compound Analyzed, Dosage and Route of Administration	AA Mediator Assessed	Results
1	Bendvold et al., 1985 [68]	Human study/*n* = 6 before, during and after treatment with naproxen	Naproxen 250 mg 3 times daily for 2 weeks; Oral administration	PGE, PGF, 19-hydroxy-PGE, and 19-hydroxy-PGF, 8α -1 9-hydroxy-PGF_2α_, 8ß-19-hydroxy-PGF_1α_	In human seminal fluid, naproxen reduced the concentration of all PGs. However, no statistically significant role of naproxen was observed on sperm density, motility, or morphology. The data suggest that the reduction of PGs is not secondary to the effect of naproxen on sperm characteristics.
2	Collier et al., 1971 [69]	Human study/*n* = 4 (22–29 years old)	Aspirin 600 mg; Oral Administration	PGE_2_ and PGF_2α_	PGE_2_ and PGF_2α_ levels in human seminal fluid were reduced during treatment with aspirin. The mechanisms responsible for controlling the concentrations of PGE_2_ and PGF_2α_ in semen may be different. No further data were reported on the role of aspirin in sperm motility, density.
3	Horton et al., 1973 [70]	Human study/*n* = 2 (25 and 52 years old respectively)	Aspirin 3.6–7.2 g/day; Oral administration	PGE	The seminal plasma levels of PGE were reduced by 80% in patients taking 7.2 g/day of Aspirin. Sperm density was not assessed.
4	Barbosa 2020 [81]	Animal Study/Male rats (23 days old)	Ibuprofen 0; 2.4; 7.2 or 14.3 mg/kg/day; Oral administration	PGs	The pre-pubertal treatment with ibuprofen had a negative effect in sperm quality and quantity affecting the reproduction. The male offspring had an accelerated sperm transit time in the epididymis, while the fertility potential reduced in the female offspring.
5	Löscher et al., 1988 [84]	Animal Study/Male rabbits	Phenylbutazone 100 mg/kg; Subcutaneous injection	PGE_2_, PGF_2α_	The prolonged treatment with NSAIDs did not affect rabbit male fertility. However, chronic treatment with phenylbutazone may improve sperm quality, increase ejaculate volume, and improve sperm fertility.
6	Löscher et al., 1986 [85]	Animal Study/Male rats	Acetylsalicylic acid 50 or 150 mg; Naproxen 10 mg; Indomethacin 2 mg; Phenylbutazone 20 mg; Intraperitoneal injection	PGE_2_	The reduction of prostaglandin synthesis in male rats does not have any affect in fertility. This can be explained by the very low seminal prostaglandin levels in rats in confront to other animals.
7	Conte et al., 1985 [86]	Human study/*n* = 15 fertile men (20–30 years); *n* = 20 infertile oligozoospermic men (20–40 years); *n* = 10 infertile oligozoospermic patients (20–40 years)	Indomethacin (100 mg/die) for 30 days; Oral administration	PGE, 19-OH PGE	Indomethacin improved significantly the sperm count and motility in infertile oligozoospermic patients with high levels of PGs
8	Yegnanarayan et al., 1978 [87]	Animal Study/Male rats	Acetyl salicylic acid, indomethacin, oxyphenbutazone 4 mg/kg, 100 mg/kg, 400 mg/kg for short term experiments; and one fifth of the above-mentioned doses for long-term experiments; Oral administration	PGE_2_, PGF_2α_	The number of fertile coatings or pregnancies was significantly less in the groups mated with indomethacin and oxyphenbutazone treated males.
9	Freixa et al., 1984 [83]	Human study/*n* = 6 (20–25 years old)	Flurbiprofen 100 mg; Oral administration	PGE, 19-OH PGEs	Flurbiprofen reduced PGE values, and produces a small alteration in sperm head with a larger and spherical head.
10	Fouchécourt et al., 2002 [27]	Animal Study/Rats	Flurbiprofen 3.3 mg/kg, Indomethacin 1.7 mg/kg; Intraperitoneal injection	PGE_2_, PGF_2_	Flurbiprofen and Indomethacin did not affect male reproduction in rats.
11	Cenedella et al., 1973 [79]	Animal Study/Mice	Aspirin (50 mg/kg twice daily); Oral administration	PGs	Interestingly the fertility increased in male mice that were classified initially as sub-fertile under treatment with aspirin (50 mg/kg twice daily for a total of 12 days).
12	Vyas et al., 2016 [71]	Animal Study/Male rats	Aspirin 12.5 mg/kg for 30 days and 60 days; Oral administration	PGs	The subchronic dose of aspirin (12.5 mg/kg for 30 days and 60 days) given to male rats changed the reproductive profile of male rats, and reduced sperm mobility and density.
13	Stutz et al., 2004 [77]	Human study/*n* = 277	Aspirin 1–8 g/mo; Oral administration	PGs	Aspirin can have a deleterious effect on seminal parameters. In moderate aspirin users the percentages of motile, progressive and rapid progressive gametes decreased.
14	Kennedy et al., 2003 [72]	Animal Study/White Turkeys	Diclofenac, Indomethacin, Aspirin, Tolmetin 0–15 mM; In-vitro	PGE_1_, PGE_2_, PGF_2α_	The NSAIDs studied decreased the avian mobility of sperm.
15	Martini et al., 2003 [78]	Human study/20–60 years old	Aspirin 2600 mg/day for 3 days; Oral administration	PGs	The chronic use of Aspirin has a negative impact on male fertility, specifically on sperm motility, morphology, and seminal volume.
16	Tanyildizi et al., 2003 [73]	Animal Study/Rams	Aspirin 75 mg kg^−1^ body weight; Oral administration/Metamizol t 50 mg kg^−1^ body weight; Intramuscular injection	PGs	The concomitant use of two NSAIDs, respectively Aspirin and Metamizol decreased both the semen volumes, and sperm concentrations.
17	Stutz et al., 2000 [82].	Animal Study/Mice	Aspirin 14.3 mg/kg day^−1^ Intramuscular injection/Ibuprofen 5.6 mg/kg day^−1^, Intraperitoneal injection /Piroxicam 0.28 mg/kg day^−1^, Intraperitoneal injection	PGs	Ibuprofen reduced testosterone levels, but did not modify the sperm functional activity. Aspirin reduced the percentage of gametes that were swelled, and irreversibly alters structural and/or functional properties of sperm plasma membrane.
18	Didolkar et al., 1980 [74]	Animal Study/Rats	Aspirin 5 mg/100 g body weight/day; Oral administration	PGs	Aspirin used for a long period of time can impair spermatogenesis and brings to an increase of plasma LH levels.
19	Didolkar et al., 1980 [75]	Animal Study/Rats	Aspirin 5 mg/100 g body weight/day for 30 days; Oral administration	PGs	Aspirin can have a deleterious effect on seminal parameters, bringing to impairment of spermatogenesis.
20	Biswas et al., 1978 [76]	Animal Study/Rats	Aspirin 5 mg/100 g body weight/day; Intraperitoneal injection	PGE_2_	The spermatid count was decreased following treatment with Aspirin.
21	Scott et al., 1978 [80]	Animal Study/Rats	Aspirin 300 mg/kg body weight, 150 mg/kg body weight) for 12 days and 6 days; Oral administration	PGs	Data did not conclude on the role of Aspirin on spermatogenesis.

## 4. Discussion

Our research study included 17 studies carried out in animals, 31 studies conducted in humans, and 21 studies that reported the association between NSAIDs and seminal fluid PGs. One of the articles was inserted in both Table 1 and Table 3, respectively.

Various evidence has shown that the AA pathway and its mediators are involved in male fertility. Starting from AA, studies demonstrated that AA itself can mediate the stimulatory effect of luteinizing hormone on the synthesis of testicular steroids [88,89]. PLA_2_ enzyme is also a major component in sperm [90]. COX-2 is expressed in testes of infertile men, or men with impaired spermatogenesis [18].

Human seminal fluid contains different prostaglandins that originate from the seminal vesicles, and play important roles in sperm function, and motility. They can act directly on spermatozoa through the PG receptors [53,91], and have a protective role in sperm motility. In particular, 19-OH-PGE has a protective role on the sperm from immunological damage [4]. PGs can also decrease the plasma concentration of androgens.

PGE is one of the main PGs assessed in male fertility in both animal and human clinical studies, suggesting that the semen of infertile men has lower levels of PGE [53]. Based on these findings, an increase of PGE levels in men, by using PGE agonist could be an option to treat male infertility. However other contradictory studies suggest that 19-OH-PGE and PGE concentrations can vary.

Despite PGE, hematopoietic PGD_2_ synthase is also expressed in patients with impaired spermatogenesis. Its levels are reduced in oligozoospermic men [92]. Analogously another PG, respectively 15d-PGJ_2_ was found in patients with idiopathic infertility [93].

Targeting the AA pathway has emerged as an attractive strategy for the treatment of male fertility. Ibuprofen can affect fertility through the alteration of sperm motility, function, viability, count potentially through the reduction of PGS synthesis [57,94,95,96].

In addition, other NSAIDs such as flurbiprofen, acetylsalicylic acid, naproxen can decrease PG levels in human semen and increase fertility [68,69,70,83]. These findings can be partially explained by Isidori et al., that high baseline levels of PGs are harmful because they reduce testicular DNA synthesis and cause down regulation of the receptors for the same PGs. Consequently, if NSAIDs are administered to subjects with too high levels of PGs, they can have a positive effect on semen quality. Indomethacin used in oligospermic men increased fertility [97], and induces alteration of endocrine system in fetal testis, together with Aspirin and Paracetamol [98]. In addition, in a mice model Indomethacin (5 mg/kg/day), decreased the fertility of mice, in contrary with lower doses of indomethacin (3 mg/kg/day) that did not have any effect in fertility [99]. Prolonged use of indomethacin (2 mg/kg twice daily for 7 days) in male rats reduced the fertility [85].

Chronic treatment (50 mg/kg twice daily for a total of 12 days) with acetylsalicylic acid in male mice [79], or treatment with naproxen (with a maximum dose of 30 mg/kg/day for 60 days) did not have any role in fertility [100]. However, interestingly, the fertility increased in male mice that were classified initially as sub-fertile under treatment with aspirin (50 mg/kg twice daily for a total of 12 days) [79].

The subchronic dose of aspirin (12.5 mg/kg for 30 days and 60 days) given to male rats changed the reproductive profile of male rats, and reduced sperm mobility and density [71]. In line with these findings, Stutz et al. confirmed that Aspirin can have a deleterious effect on seminal parameters [77].

## 5. Conclusions

To our knowledge, this is the first study reporting and evaluating all the published studies on the association of AA pathways mediators and male fertility. Of notice, most of the references are old, and only few studies have been performed recently in the field. Studies reported in this article are not homogeneous, and often report conflicting results. However, we believe that based on the promising results from either animal or human studies, it is the duty of the academic world to keep exploring the AA pathway’s implication in male fertility. Considering that PGE is the main PG involved in male fertility, and its levels are decreased in the semen of infertile men, the PGE agonist, or any drug which causes an increase in seminal PGE concentration could be suggested as a potential approach to improve male fertility. In addition, we notice that there is no information on the role of leuokotrienes, or lipoxins in semen, and on the role of sperm motility, morphology, function, and fertility. Additional studies should be carried out to further explore other AA pathways mediators, or enzymes. In addition, long term effect of NSAIDs in male fertility should be further explored. Considering that COX-2 is expressed in the testes of infertile men and is implicated in testicular inflammation related to idiopathic infertility it would be interesting assessing also the role of COXIBs in male fertility.

We believe that despite some controversial results targeting the AA pathway is a promising strategy to be further explored for expanding treatment options for male infertility.

## Data Availability

Not applicable.

## References

[1] Capra V., Rovati G.E., Mangano P., Buccellati C., Murphy R.C., Sala A. (2015). Transcellular biosynthesis of eicosanoid lipid mediators. Biochim. Biophys. Acta.

[2] Von Euler U.S. (1936). On the specific vaso-dilating and plain muscle stimulating substances from accessory genital glands in man and certain animals. J. Physiol..

[3] Kelly R.W. (1981). Prostaglandin synthesis in the male and female reproductive tract. J. Reprod. Fertil..

[4] Templeton A.A., Cooper I., Kelly R.W. (1978). Prostaglandin concentrations in the semen of fertile men. J. Reprod. Fertil..

[5] Bendvold E., Gottlieb C., Svanborg K., Bygdeman M., Eneroth P. (1987). Concentration of prostaglandins in seminal fluid of fertile men. Int. J. Androl..

[6] Gerozissis K., Jouannet P., Soufir J.C., Dray F. (1982). Origin of prostaglandins in human semen. J. Reprod. Fertil..

[7] Cosentino M.J., Hastings N.E., Ellis L.C. (1982). Prostaglandins and cyclic nucleotides in the ram reproductive tract. J. Androl. (Abstr.).

[8] Ollero M., Powers R.D., Alvarez J.G. (2000). Variation of docosahexaenoic acid content in subsets of human spermatozoa at different stages of maturation: Implications for sperm lipoperoxidative damage. Mol. Reprod. Dev..

[9] Ollero M., Gil-Guzman E., Lopez M.C., Sharma R.K., Agarwal A., Larson K., Evenson D., Thomas A.J., Alvarez J.G. (2001). Characterization of subsets of human spermatozoa at different stages of maturation: Implications in the diagnosis and treatment of male in-fertility. Hum. Reprod..

[10] Aitken R.J., Wingate J.K., De Iuliis G.N., Koppers A.J., McLaughlin E.A. (2006). *Cis*-Unsaturated Fatty Acids Stimulate Reactive Oxygen Species Generation and Lipid Peroxidation in Human Spermatozoa. J. Clin. Endocrinol. Metab..

[11] Hedqvist P., Gustafsson L., Hjemdahl P., Svanborg K., Samuelsson B., Ramwell P., Paoletti R. (1980). Aspects of prostaglandin action on autonomic neuroeffector transmis-sion. Advances in Prostaglandin and Thromboxane Research.

[12] Peterson R.N., Seyler B., Bundman D., Freund M. (1980). The effect oftheophylline and dibutyryl cyclic AMP on the uptake of radio-active calcium and phosphate ions by boar and human spermatozoa. J. Reprod. Fertil..

[13] Cosentino M.J., Emilson L.B., Cockett A.T. (1984). Prostaglandins in semen and their relationship to male fertility: A study of 145 men. Fertil. Steril..

[14] Cheuk B.L.Y., Chew S.C., Fiscus R.R., Wong P.Y.D. (2002). Cyclooxygenase-2 regulates apoptosis in rat epididymis through prostaglandin D2. Biol. Reprod..

[15] Olsson J.E. (1975). Correlation between the concentration of β-trace protein and the number of spermatozoa in human semen. J. Reprod. Fertil..

[16] Leone M.G., Haq H.A., Saso L. (2002). Lipocalin type prostaglandin D-synthase: Which role in male fertility?. Contraception.

[17] Chen D.-Y., Zhu M.-Y., Cui Y.-D., Huang T.-H. (2007). Relationship between contents of lipocalin-type prostaglandin D synthase on the surface of infertility sperm and in seminal plasma. Biochemistry.

[18] Frungieri M.B., Calandra R.S., Mayerhofer A., Matzkin M.E. (2015). Cyclooxygenase and prostaglandins in somatic cell populations of the testes. Reproduction.

[19] Rudolph L.M., Bentley G.E., Calandra R.S., Paredes A.H., Tesone M., Wu T.-Y., Micevych P.E. (2016). Peripheral and Central Mechanisms Involved in the Hormonal Control of Male and Female Reproduction. J. Neuroendocrinol..

[20] Hase T., Yoshimura R., Matsuyama M., Kawahito Y., Wada S., Tsuchida K., Sano H., Nakatani T. (2003). Cyclooxygenase-1 and -2 in human testicular tumours. Eur. J. Cancer..

[21] Schell C., Frungieri M.B., Albrecht M., Gonzalez-Calvar S.I., Ko¨hn F.M., Calandra R.S., Mayerhofer A. (2007). A prostaglandin D2 system in the human testis. Fertil. Steril..

[22] Szczuko M., Kikut J., Komorniak N., Bilicki J., Celewicz Z., Ziętek M. (2020). The Role of Arachidonic and Linoleic Acid De-rivatives in Pathological Pregnancies and the Human Reproduction Process. Int. J. Mol. Sci..

[23] Lax Y., Grossman S., Rubinstein S., Magid N., Breitbart H. (1990). Role of lipoxygenase in the mechanism of acrosome reaction in mammalian spermatozoa. Biochim. Et Biophys. Acta (BBA)-Lipids Lipid Metab..

[24] Olive E.H., Fabiani R., Johansson L., Ronquist G. (1993). Arachidonic acid 15-lipoxygenase and traces of E prostaglandins in pu-rified human prostasomes. J. Reprod. Fertil..

[25] Bao S., Miller D.J., Ma Z., Wohltmann M., Eng G., Ramanadham S., Moley K., Turk J. (2004). Male Mice that Do Not Express Group VIA Phospholipase A2 Produce Spermatozoa with Impaired Motility and Have Greatly Reduced Fertility. J. Biol. Chem..

[26] Waheed M., Ghoneim I., Alhaider A. (2014). Seminal plasma and serum fertility biomarkers in dromedary camels (*Camelus dromedarius*). Theriogenology.

[27] Fouchécourt S., Charpigny G., Reinaud P., Dumont P., Dacheux J.-L. (2002). Mammalian Lipocalin-Type Prostaglandin D2 Synthase in the Fluids of the Male Genital Tract: Putative Biochemical and Physiological Functions. Biol. Reprod..

[28] Hale B.J., Fernandez R.F., Kim S.Q., Diaz V.D., Jackson S.N., Liu L., Brenna J.T., Hermann B.P., Geyer C.B., Ellis J.M. (2019). Acyl-CoA syn-thetase 6 enriches seminiferous tubules with the ω-3 fatty acid docosahexaenoic acid and is required for male fertility in the mouse. J. Biol. Chem..

[29] Stoffel W., Schmidt-Soltau I., Binczek E., Thomas A., Thevis M., Wegner I. (2020). Dietary ω3- and ω6-Polyunsaturated fatty acids reconstitute fertility of Juvenile and adult Fads2-Deficient mice. Mol. Metab..

[30] Roqueta-Rivera M., Stroud C.K., Haschek W.M., Akare S.J., Segre M., Brush R.S., Agbaga M.-P., Anderson R.E., Hess R.A., Nakamura M.T. (2010). Docosahexaenoic acid supplementation fully restores fertility and spermatogenesis in male delta-6 desaturase-null mice. J. Lipid Res..

[31] Khatibjoo A., Kermanshahi H., Golian A., Zaghari M. (2018). The effect of n-6/n-3 fatty acid ratios on broiler breeder performance, hatchability, fatty acid profile and reproduction. J. Anim. Physiol. Anim. Nutr..

[32] Gerozissis K., Dray F. (1981). Radioimmunoassay of prostaglandins in the semen of fertile men. J. Reprod. Fertil..

[33] Castracane V.D., Saksena S.C. (1974). Prostaglandins of E series and LH release in fertile male rats. Prostaglandins.

[34] Memon G.N. (1973). Effects of intratesticular injections of prostaglandins on the testes and accessory sex glands of rats. Contraception.

[35] Rej S.K., Chatterjee A. (1980). The possible mode of action of prostaglandins: XVI. A study to assess the local effect of prostaglandins E1, E2 or F2α in the regulation of male fertility. Prostaglandins Med..

[36] Bartke A., Iusto N., Caldwell B.V., Behrman H.R. (1973). Effects of a cholesterol inhibitor and of prostaglandin F2α on testis cholesterol and on plasma testosterone in mice. Prostaglandins.

[37] Lubice-Nawrocki C.M., Saksena S.K., Chang M.C. (1973). Effects of prostaglandins El and F2α on the fertilizing ability of hamster spermatozoa. J. Reprod. Fert..

[38] Ericsson R.J. (1973). Prostaglandins (E1 and E2) and reproduction in the male rat. Adv. Biosci..

[39] Hafiez A.A. (1974). Prostaglandin E2 prevents impairment of fertility in rats fed a diet deficient in essential fatty acids. J. Reprod. Fertil..

[40] Kimball F.A., Frielink R.D., Porteus S.E. (1978). Effects of 15(s)-15-methyl prostaglandin F2A methyl ester-containin silastic disc in male rats. Fertil. Steril..

[41] Free M.J., Jaffe R.A. (1972). Effect of prostaglandins on blood flow and pressure in the conscious rat. Prostaglandins.

[42] Isidori A., Conte D., Laguzzi G., Giovenco P., Dondero F. (1980). Role of seminal prostaglandins in male fertility. I. Relationship of prostaglandin E and 19-OH prostaglandin E with seminal parameters. J. Endocrinol. Investig..

[43] Tusell J.M., Gelpi E. (1980). Prostaglandins E and F, and 19-hydroxylated E and F (series I and II) in semen of fertile men. Gas and liquid chromatographic separation with selected ion detection. J. Chromatogr..

[44] Sturde H.G. (1968). Experimental investigations into the question of prostaglandins and their relation to male fertility. Parts I, II, III. Arzneim. Forsch..

[45] Shrivastav P., Swann J., Jeremy J.Y., Thompson C., Shaw R.W., Dandona P. (1989). Sperm Function and Structure and Seminal Plasma Prostanoid Concentrations in Men with IDDM. Diabetes Care.

[46] Andersen J.M., Rønning P.O., Herning H., Bekken S.D., Haugen T.B., Witczak O. (2016). Fatty acid composition of spermatozoa is associated with BMI and with semen quality. Andrology.

[47] Safarinejad M.R., Hosseini Y., Dadkhah F., Asgari M.A. (2010). Relationship of omega-3 and omega-6 fatty acids with semen characteristics, and anti-oxidant status of seminal plasma: A comparison between fertile and infertile men. Clin. Nutr..

[48] Hawkins D.F., Labrum A.H. (1961). Semen prostaglandin levels in fifty patients attending a fertility clinic. J. Reprod. Fertil..

[49] Lewy R.I., Bills T.K., Dalton J., Smith J.P., Silver M.J. (1979). 19-hydroxy-prostaglandin E and infertility in human males. Prostaglandins Med..

[50] Horton E.W., Thompson C.J. (1964). Thin-layer chromatography and bioassay of prostaglandins in extracts of semen and tissues of the male reproductive tract. Br. J. Pharmacol. Chemother..

[51] Skakkeback N.E., Kely R.W., Corter C.S. (1976). Prostaglandin concentrations in the semen of hypogonadal men during treatment with testosterone. J. Reprod. Fert..

[52] Bygdeman M., Fredricsson B., Svanborg K., Samuelsson B. (1970). The Relation Between Fertility and Prostaglandin Content of Seminal Fluid in Man. Fertil. Steril..

[53] Collier J.G., Flower R.J., Stanton S.L. (1975). Seminal prostaglandins in infertile men. Fertil. Steril..

[54] Brummer H.C., Gillespie A. (1972). Seminal prostaglandins and fertility. Clin. Endocrinol..

[55] Huleihel M., Lunenfeld E., Horowitz S., Levy A., Potashnik G., Mazor M., Glezerman M. (1999). Expression of IL-12, IL-10, PGE2, sIL-2R and sIL-6R in seminal plasma of fertile and infertile men. Andrologia.

[56] Moretti E., Collodel G., Salvatici M.C., Belmonte G., Signorini C. (2019). New insights into sperm with total globozoospermia: In-creased fatty acid oxidation and centrin1 alteration. Syst. Biol. Reprod. Med..

[57] Gottlieb C., Svanborg K., Eneroth P., Bygdeman M. (1988). Effect of prostaglandins on human sperm function in vitro and seminal adenosine triphosphate content. Fertil. Steril..

[58] Schell C., Albrecht M., Spillner S., Mayer C., Kunz L., Kohn F.M., Schwarzer U., Mayerhofer A. (2010). 15-Deoxy-Δ12-14-Prostaglandin-J2 Induces Hypertrophy and Loss of Contractility in Human Testicular Peritubular Cells: Implications for Human Male Fertility. Endocrinology.

[59] Longini M., Moretti E., Signorini C., Noto D., Iacoponi F., Collodel G. (2020). Relevance of seminal F2-dihomo-IsoPs, F2-IsoPs and F4-NeuroPs in idiopathic infertility and varicocele. Prostaglandins Other Lipid Mediat..

[60] Collodel G., Noto D., Signorini C., Gambera L., Stendardi A., Mahmutbegovic A., Micheli L., Menchiari A., Moretti E. (2021). Do Seminal Isoprostanes Have a Role in Assisted Reproduction Outcome?. Life.

[61] Collodel G., Moretti E., Longini M., Pascarelli N.A., Signorini C. (2018). Increased F2-Isoprostane Levels in Semen and Immuno-localization of the 8-Iso Prostaglandin F2α in Spermatozoa from Infertile Patients with Varicocele. Oxid. Med. Cell. Longev..

[62] Moretti E., Signorini C., Ferretti F., Noto D., Collodel G. (2022). A Study to Validate the Relevance of Semen F2-Isoprostanes on Human Male Infertility. Int. J. Environ. Res. Public Health.

[63] Signorini C., Moretti E., Noto D., Micheli L., Ponchia R., Collodel G. (2022). Fatty Acid Oxidation and Pro-Resolving Lipid Mediators Are Related to Male Infertility. Antioxidants.

[64] Asplund J. (1947). A Quantitative Determination of the Content of Contractive Substances in Human Sperm and their Significance for the Motility and Vitality of the Spermatozoa. Acta Physiol. Scand..

[65] Kelly R.W., Cooper I., Templeton A.A. (1979). Reduced prostaglandin levels in the semen of men with very high sperm concen-trations. J. Reprod. Fertil..

[66] Dorp J. (1968). Essential fatty acids and prostaglandin. Nat. Sci..

[67] Schlegel W., Rotermund S., Färber G., Nieschlag E. (1981). The influence of prostaglandins on sperm motility. Prostaglandins.

[68] Bendvold E., Gottlieb C., Svanborg K., Bygdeman M., Eneroth P., Cai Q.-H. (1985). The effect of naproxen on the concentration of prostaglandins in human seminal fluid. Fertil. Steril..

[69] Collier J.G., Flower R.J. (1971). Effect of aspirin on human seminal prostaglandins. Lancet.

[70] Horton E.W., Jones R.L., Marr C.G. (1973). Effects of aspirin on prostaglandin and fructose levels in human semen. J. Reprod. Fertil..

[71] Vyas A., Ram H., Purohit A., Jatwa R. (2016). Adverse effects of subchronic dose of aspirin on reproductive profile of male rats. J. Pharm..

[72] Kennedy J.H., Korn N., Thurston R.J. (2003). Prostaglandin levels in seminal plasma and sperm extracts of the domestic turkey, and the effects of cyclooxygenase inhibitors on sperm mobility. Reprod. Biol. Endocrinol..

[73] Tanyıldızı S., Bozkurt T. (2003). Effects of acetylsalicylic acid and metamizol on hyaluronidase activity and sperm characteristics in rams. Anim. Reprod. Sci..

[74] Didolkar A.K., Gurjar A., Joshi U.M., Sheth A.R., Roychowdhury D. (1980). Effects of aspirin on blood plasma levels of testosterone, LH and FSH in maturing male rats. Int. J. Androl..

[75] Didolkar A.K., Patel P.B., Roychowdhury D. (1980). Effect of aspirin on spermatogenesis in mature and immature rats. Int. J. Androl..

[76] Biswas N.M., Sanyal S., Patra P.B. (1978). Antispermatogenic Effect of Aspirin and its Prevention by Prostaglandin E2. Andrologia.

[77] Stutz G., Zamudio J., Santillán M.E., Vincenti L., De Cuneo M.F., Ruiz R.D. (2004). The Effect of Alcohol, Tobacco, and Aspirin Consumption on Seminal Quality among Healthy Young Men. Arch. Environ. Health.

[78] Martini A.C., Molina R.I., Tissera A.D., Ruiz R.D., Fiol de Cuneo M. (2003). Analysis of semen from patients chronically treated with low or moderate doses of aspirin-like drugs. Fertil. Steril..

[79] Cenedella R.J., Crouthamel W.G. (1973). Effect of aspirin upon male mouse fertility. Prostaglandins.

[80] Scott J.E., Persaud T.V. (1978). A quantitative study of the effects of acetylsalicylic acid on spermatogenesis and organs of the rat. Int. J. Fertil..

[81] Barbosa M.G., Jorge B.C., Stein J., Ferreira D.A.S., Barreto A.C.D.S., Reis A.C.C., Moreira S.D.S., Inocencio L.C.D.L., Macorini L.F.B., Arena A.C. (2020). Pre-pubertal exposure to ibuprofen impairs sperm parameters in male adult rats and compromises the next generation. J. Toxicol. Environ. Health Part A.

[82] Stutz G., Martini A.C., Ruiz R.D., Fiol De Cuneo M., Munoz L., Lacuara J.L. (2000). Functional activity of mouse sperm was not affected by low doses of aspirin-like drugs. Arch. Androl..

[83] Freixa R., RosellóCatafau J., Gelpí E., Iglesias Cortit J.L., Ballescá J.L., de Paz J.L., Iglesias Guiu J., Gonzalez Merlo J., Puig Parellada P. (1984). Comparative study of antiinflammatory drugs and sulphasalazine in relation to prostaglandin E and 19 hydroxylated prostaglandin E levels and human male fertility. Prostaglandins Leukot. Med..

[84] Löscher W., Lüttgenau H., Schlegel W., Krüger S. (1988). Pharmacokinetics of non-steroidal anti-inflammatory drugs in male rabbits after acute and chronic administration and effect of chronic treatment on seminal prostaglandins, sperm quality and fertility. J. Reprod. Fertil..

[85] Loscher W., Blazaki D. (1986). Effect of non-steroidal anti-inflammatory drugs on fertility of male rats. J. Reprod. Fertil..

[86] Conte D., Nordio M., Ronnanelli F., Manganelli F., Giovenco P., Dondero F., Isidori A. (1985). Role of seminal prostaglandins in male fertility. II. Effects of prostaglandin synthesis inhibition on spermatogenesis in man. J. Endocrinol. Investig..

[87] Yegnanarayan R., Joglekar G. (1978). Anti-Fertility Effect of Non-Steroidal Anti-Inflammatory Drugs. Jpn. J. Pharmacol..

[88] Abayasekara D., Wathes D. (1999). Effects of altering dietary fatty acid composition on prostaglandin synthesis and fertility. Prostaglandins Leukot. Essent. Fat. Acids.

[89] Didolkar A.K., Sunderam K. (1987). Arachidonic acid is involved in the regulation of hCG-induced steroidogenesis in rat Leydig cells. Life Sci..

[90] Roldan E.R., Shi Q.X. (2007). Sperm phospholipases and acrosomal exocytosis. Front. Biosci..

[91] Mercado E., Villalobos M., Domínguez R., Rosado A. (1978). Differential binding of PGE-1 and PGF-2α to the human spermatozoa membrane. Life Sci..

[92] Tokugawa Y., Kunishige I., Kubota Y., Shimoya K., Nobunaga T., Kimura T., Saji F., Murata Y., Eguchi N., Oda H. (1998). Lipocalin-type prostaglandin D synthase in human male reproductive organs and seminal plasma. Biol. Reprod..

[93] Kampfer C., Spillner S., Spinnler K., Schwarzer J.U., Terradas C., Ponzio R., Puigdomenech E., Levalle O., Köhn F.M., Matzkin M.E. (2012). Evidence for an adaptation in ROS 647 scavenging systems in human peritubular testicular cells from infertility 648 patients. Int. J. Androl..

[94] Banihani S.A. (2019). Effect of ibuprofen on semen quality. Andrologia.

[95] Kristensen D.M., Hass U., Lesné L., Lottrup G., Jacobsen P.R., Desdoits-Lethimonier C., Boberg J., Petersen J.H., Toppari J., Jensen T.K. (2011). Intrauterine exposure to mild analgesics is a risk factor for development of male reproductive disorders in human and rat. Hum. Reprod..

[96] Lind D.V., Main K.M., Kyhl H.B., Kristensen D.M., Toppari J., Andersen H.R., Andersen M.S., Skakkebæk N.E., Jensen T.K. (2017). Maternal use of mild analgesics during pregnancy associated with reduced anogenital distance in sons: A cohort study of 1027 mother-child pairs. Hum. Reprod..

[97] Barkay J., Harpaz-Kerpel S., Ben-Ezra S., Gordon S., Zuckerman H. (1984). The prostaglandin inhibitor effect of antiinflammatory drugs in the therapy of male infertility. Fert. Steril..

[98] Mazaud-Guittot S., Nicolaz C.N., Desdoits-Lethimonier C., Coiffec I., Ben Maamar M., Balaguer P., Kristensen D.M., Chevrier C., Lavoué V., Poulain P. (2013). Paracetamol, Aspirin, and Indomethacin Induce Endocrine Disturbances in the Human Fetal Testis Capable of Interfering with Testicular Descent. J. Clin. Endocrinol. Metab..

[99] Marley P.B., Smith C.C. (1974). Proceedings: The source and a possible function in fertility of seminal prostaglandin-like material, in the mouse. Br. J. Pharmacol..

[100] Hallesy D.W., Short L.D., Hill R. (1973). Comparative toxicology of naproxen. Scand. J. Rheumatol..

